# Xenobiotic metabolism in differentiated human bronchial epithelial cells

**DOI:** 10.1007/s00204-016-1868-7

**Published:** 2016-10-13

**Authors:** Jan J. W. A. Boei, Sylvia Vermeulen, Binie Klein, Pieter S. Hiemstra, Renate M. Verhoosel, Danyel G. J. Jennen, Agustin Lahoz, Hans Gmuender, Harry Vrieling

**Affiliations:** 1 Department of Human Genetics, Leiden University Medical Center, Postal Zone S4-P, PO Box 9600, 2300 RC Leiden, The Netherlands; 2 Department of Pulmonology, Leiden University Medical Center, 2300 RC Leiden, The Netherlands; 3 Department of Toxicogenomics, Maastricht University, Maastricht, The Netherlands; 4 Unidad de Hepatología Experimental, Instituto de Investigación Sanitaria-Fundación Hospital La Fe, 46009 Valencia, Spain; 5 Genedata AG, 4053 Basel, Switzerland

**Keywords:** Lung, Metabolic activity, Gene expression profiling, Bronchial epithelial cells, Cytochrome P450

## Abstract

Differentiated human bronchial epithelial cells in air liquid interface cultures (ALI-PBEC) represent a promising alternative for inhalation studies with rodents as these 3D airway epithelial tissue cultures recapitulate the human airway in multiple aspects, including morphology, cell type composition, gene expression and xenobiotic metabolism. We performed a detailed longitudinal gene expression analysis during the differentiation of submerged primary human bronchial epithelial cells into ALI-PBEC to assess the reproducibility and inter-individual variability of changes in transcriptional activity during this process. We generated ALI-PBEC cultures from four donors and focussed our analysis on the expression levels of 362 genes involved in biotransformation, which are of primary importance for toxicological studies. Expression of various of these genes (e.g., GSTA1, ADH1C, ALDH1A1, CYP2B6, CYP2F1, CYP4B1, CYP4X1 and CYP4Z1) was elevated following the mucociliary differentiation of airway epithelial cells into a pseudo-stratified epithelial layer. Although a substantial number of genes were differentially expressed between donors, the differences in fold changes were generally small. Metabolic activity measurements applying a variety of different cytochrome p450 substrates indicated that epithelial cultures at the early stages of differentiation are incapable of biotransformation. In contrast, mature ALI-PBEC cultures were proficient in the metabolic conversion of a variety of substrates albeit with considerable variation between donors. In summary, our data indicate a distinct increase in biotransformation capacity during differentiation of PBECs at the air–liquid interface and that the generation of biotransformation competent ALI-PBEC cultures is a reproducible process with little variability between cultures derived from four different donors.

**Electronic supplementary material:**

The online version of this article (doi:10.1007/s00204-016-1868-7) contains supplementary material, which is available to authorized users.

## Introduction

Inhalation represents one of the major routes by which humans are exposed to gases, volatile compounds, aerosols and respirable particles. After entrance into the respiratory tract, inhaled materials may be absorbed by pulmonary cells and surrounding tissues. Cells of the respiratory tract have been shown to be metabolically competent and able to biotransform a large variety of inhaled substances (Anttila et al. 2011; Hukkanen et al. 2002; Zhang et al. 2006). These biotransformation reactions enable detoxification of xenobiotics and facilitate elimination of hazardous compounds from the body (Nebert and Dalton 2006). However, in some cases biotransformation results in the formation of highly reactive intermediates that can bind to various biomolecules including DNA. A classic example is the activation of benzo(a)pyrene, a constituent of tobacco smoke and combustion products, into reactive species leading to the formation of mutagenic DNA adducts that have been implicated in the development of cancer (Denissenko et al. 1996).

Assessment of the potential cancer risk of substances for man is still largely based on data obtained from studies with laboratory animals. While extensive toxicological data have been generated using in vivo animal models exposed via oral or dermal administration, the amount of data obtained using inhalation as an exposure route is far more limited. The high workload, the requirement of specialized equipment and knowledge, and high costs are among the main practical barriers for inhalation studies. Additionally, extrapolation of lung cancer data obtained with experimental animals to humans is inherently difficult and not only because of physiological differences. Various carcinogenic airborne substances show species-dependent differences with regard to their potential to induce tumors that for a significant part can be attributed to species-related differences in biotransforming capacity within the lung (Bond and Medinsky 2001; Cruzan et al. 2009). These observations raise concerns about the suitability of animal exposures for health risk prediction of airborne substance in humans.

The development of sophisticated in vitro models based on cells from human pulmonary origin might contribute to a better understanding of the basis of human lung toxicity and might ultimately form an alternative for inhalation studies with laboratory animals. In recent years, protocols have been established for the generation of air–liquid interface (ALI) cultures of well-differentiated airway epithelial cells containing various cell-types including club (formerly called Clara cells), ciliated and goblet cells. The air-exposed nature of these cultures makes them promising vehicles for toxicity studies of airborne substances (Iskandar et al. 2015; Kogel et al. 2015; Mathis et al. 2013; Neilson et al. 2015). Even chronic long-term or repeated exposures, to better mimic human occupational exposures, are realistic possibilities (Talikka et al. 2014). Furthermore, use of cells from designated patient populations or exposure of the cells during differentiation to disease-specific factors, such as Th2 cytokines for allergic airways inflammation, can be used to generate disease-specific models. Toxicity can be assessed applying conventional cytotoxic end points such as the inhibition of cell proliferation and the induction of cell death, as well as on the tissue level by monitoring changes in its composition, ciliary beating frequency and barrier function [e.g., changes in transepithelial electrical resistance (TEER)] (Kuehn et al. 2015). For these purposes, it is important that these ALI cultures mimic human lung epithelium in their ability to biotransform xenobiotic chemicals which is supported by the presence of differentiated cell types such as club cells that have been implicated in biotransformation processes in the lung (Chichester et al. 1991; Hukkanen et al. 2002). Transcriptome analyses showed that these organotypic cultures recapitulate the transcriptional profile of in vivo airway epithelia (Dvorak et al. 2011; Pezzulo et al. 2011) while metabolic activity of for instance CYP1A1/1B1 and CYP2A6/2A13 is conserved (Baxter et al. 2015; Newland et al. 2011). In this study, we aimed to get a better insight into the temporal changes in gene activity during the differentiation of submerged primary human bronchial epithelial cells into ALI-PBEC and to assess the reproducibility and inter-individual variability of changes in transcriptional activity during this process. Focusing on a comprehensive list of genes relevant for biotransformation processes including Phase I and II enzymes, transporter genes and nuclear receptors, we addressed the reproducibility and inter-individual variability of expression of these genes and determined the ability of these cultures to metabolize a variety of xenobiotic compounds.

## Materials and methods

### Cell culture

Primary human bronchial epithelial cells were isolated from tumor-free resected lung tissue from four anonymous donors by enzymatic digestion essentially as described previously (van Wetering et al. 2000). Cells were expanded in keratinocyte serum-free medium (KSFM, Gibco) by sub-culturing until passage 3, and aliquots were stored in liquid nitrogen. ALI-PBEC were generated essentially as previously described (Amatngalim et al. 2015; van Wetering et al. 2007). In brief, cells were brought into culture and after the next passage they were collected and seeded in transwell tissue culture inserts (Corning Costar) with a pore size of 0.4 µm and a surface of 1.12 cm^2^. Transwells were coated with a mixture of 30 mg/ml PureCol (Advanced BioMatrix, San Diego, CA), 10 μg/ml BSA (Sigma-Aldrich, St. Louis, MO) and 10 μg/ml fibronectin (isolated from human plasma) diluted in PBS, at 37 °C, 5 % CO2 for 2–24 h. Initially, cultures were grown under submerged conditions using a 1:1 mixture of DMEM (Life Technologies, Bleiswijk, the Netherlands) and bronchial epithelial growth medium (Lonza, Verviers, Belgium) (B/D medium) with supplementation of BEGM BulletKit singlequots (0.4 % [w/v] bovine pituitary extract, 1 mM hydrocortisone, 0.5 μg/ml human hEGF, 0.5 μg/ml epinephrine, 10 μg/ml transferrin, 5 μg/ml insulin, T3 and 0.1 ng/ml retinoic acid) (Lonza) and additional 1 mM HEPES (Lonza), 1 μg/ml BSA (Sigma-Aldrich), 100 U/ml penicillin and 100 μg/ml streptomycin (Lonza) and 15 ng/ml retinoic acid (Sigma-Aldrich). After around 6 days, the medium above the confluent cell layer was removed. Air-exposed cultures were maintained for up to 28 days with growth medium replacement every 2–3 days following an apical wash with 100 µl PBS to remove excess mucus. Mucociliary differentiation was typically observed between day 7 and 11 independent of the donor. Differentiated cell cultures expressed markers of basal, ciliated and goblet cells (Amatngalim et al. 2015; Mertens et al. 2016). Club cells were present in very low amounts as shown by expression of SCGB1A1 (unpublished observation). All cultures were maintained at 37 °C in a humidified atmosphere of 5 % CO_2_.

### Chemical exposure of ALI-PBEC cultures

Mature ALI-PBEC cultures, maintained at the air–liquid interface for 15 days, were exposed to 20 µM benzo[a]pyrene (B[a]P), 125 µM dibenzo(a,h)anthracene (DBA) or 50 nM 2,3,7,8,-tetrachlorodibenzo-para-dioxin (TCDD) for 24 or 72 h in comparison with time-matched solvent (DMSO) controls. All chemicals were obtained from Sigma-Aldrich, and the final DMSO concentration was 0.5 % (v/v) for all exposed cultures. Directly after exposure, RNA was isolated as described below. Measurement of trans-epithelial electrical resistance (TEER), a marker for epithelial layer disruption, using the Millicell ERS-2 (Millipore), was used to assess toxicity induced after 24-h exposure to 2 µM aflatoxin B1 with and without 72 h pretreatment to 100 nM TCDD.

### RNA isolation and microarray hybridization

Microarray analysis was performed on passage 4 primary bronchial epithelial cells grown under various conditions ranging from undifferentiated cells grown under submerged conditions to differentiated cells in air-exposed cultures. Total RNA was extracted using TRIzol (Life Technologies) according to the manufacturer’s instructions and purified using RNeasy mini kits (Qiagen). RNA purification and quality were assessed using the Agilent 2100 bioanalyzer to determine the 28S:18S rRNA ratio. Sample preparation, hybridization, washing, staining and scanning of the Affymetrix Human Genome U133 Plus 2.0 GeneChip arrays were performed as previously described (Jennen et al. 2010). The microarray data are available through the diXa data warehouse (Hendrickx et al. 2015) under study ID DIXA-004 (sample names starting with LUM-1; B[a]P, LUM-2; differentiation, LUM-4; DBA and TCDD) (http://wwwdev.ebi.ac.uk/fg/dixa/group/DIXA-004).

### Microarray data analysis

After a quality assessment step, expression values were normalized by GeneChip robust multi-array average (GC-RMA) and further analyzed with Genedata Expressionist 6.2 and GeneSpring (GX 10.0.1), respectively. In this study, we restricted the analysis to a comprehensive list of genes involved in xenobiotic metabolism. As starting point, the complete list of (Leclerc et al. 2011) was used supplemented with additional genes from the gene ontology terms 0006805 ‘xenobiotic metabolic process’ and 0017144 ‘Drug metabolic process’ and the KEGG maps 00980 ‘Metabolism of xenobiotics by cytochrome p450’ and 00982 ‘drug metabolism—cytochrome p450.’ The genes on the compiled list are represented by 779 probe sets present on the applied Affymetrix U133 Plus 2.0 microarrays. Probe sets annotated as ‘_x_at’ were excluded from the analysis as they contain probes that are identical or highly similar to unrelated sequences. In total, 679 probe sets were considered representing a total of 362 genes. The genes were subdivided into the same 5 categories as done by (Leclerc et al. 2011), namely Phase I enzymes, Phase II enzymes, transporter genes, nuclear receptor genes and a set of other genes. The distribution of the genes over the categories is shown in Table 1. For a complete list of genes and probe sets see Supplementary Table S1.

**Table 1:** Differentially expressed genes per category of biotransformation

Category	Number of genes	Number of genes with altered expression	Percentage of genes with altered expression (%)
Phase I enzymes	128	62	48.4
Phase II enzymes	52	25	48.1
Transporter	104	32	30.8
Receptor	48	14	29.2
Other	30	10	33.3
Total	362	143	39.5

BH *q* value ≤0.05 and FC ≥ 2

To identify differentially regulated genes for each culture condition, paired ANOVA analysis with donors as pairing variable and a fold-change threshold of ≥2 for the group medians was performed. The Benjamini–Hochberg (BH) procedure was applied to *p* values for multiple testing correction (Benjamini and Hochberg 1995). This procedure resulted in the identification of 143 genes (BH-corrected *q* values ≤0.05) from which the expression was significantly altered during the process of differentiation (Table 1).

### Evaluation of P450 activities

The basal activities of cytochrome p450 s were determined with a cocktail of eight substrates as described previously (Lahoz et al. 2008). The substrate mixture stock solutions was prepared in DMSO and diluted in growth medium to obtain the following final concentrations: 10 µM phenacetin (CYP1A2), 5 µM coumarin (CYP2A6), 10 µM bupropion (CYP2B6), 10 µM diclofenac (CYP2C9), 50 µM mephenytoin (CYP2C19), 10 µM bufuralol (CYP2D6), 50 µM chlorzoxazone (CYP2E1) and 5 µM midazolam (CYP3A4). The final concentration of DMSO during incubation was 0.5 % (v/v). ALI-PBEC were exposed for 5 h to the substrate cocktail in 1 ml medium. Exposure was either from below the insert (basal side), from inside the insert (apical side) or at both sides of the insert. ALI-PBEC cultures contain between 0.6 and 0.8 × 10^6^ cells. A similar number of undifferentiated submerged growing cells were incubated with the substrate cocktail. At the end of the incubation time, aliquots of the media were collected and stored at −80 °C until delivery and analysis at Analytical Unit at IIS-La Fe (Valencia, Spain). Samples were analyzed as described previously (Lahoz et al. 2008). For each condition, two independent ALI-PBEC cultures were used for the analysis. Enzymatic activities were expressed as picomole of metabolites formed per tissue culture per hour.

The induced activity of CYP1A1 and CYP1B1 after exposure to TCDD was accessed using the P450-Glo CYP1A1 assay kit (Promega) containing luciferin-CEE, a typical substrate for both CYP1A1 and CYP1B1 (for details see documentation at the Promega website). The assay was performed according to the guidelines of the manufacturer.

## Results

### Influence of differentiation on the expression of genes involved in biotransformation

**Fig. 1:**
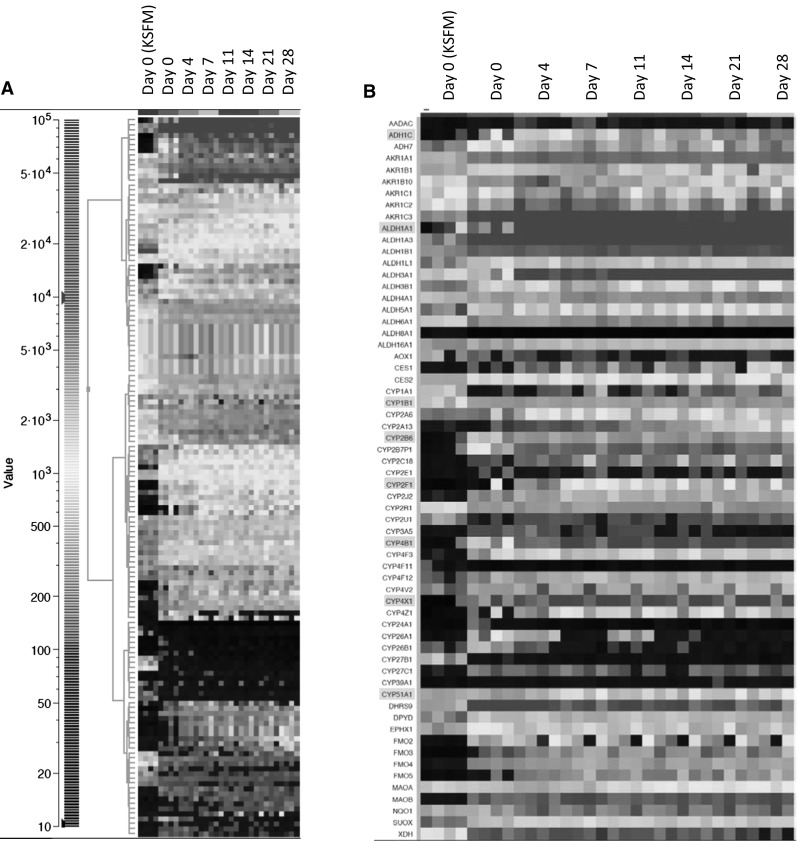
**a** 2D hierarchical clustering of the expression levels of the 143 differentially expressed genes. *Columns* are grouped according to the sampling time point. On day 0, the cells were still growing submerged either sub-confluent in KSFM medium or confluent in B/D medium. The other time points represent the duration of air-exposed culturing. The *four columns* per sampling time point represent the individual expression values for the four donors (from *left* to *right*; BR200, BR234, BR259 and BR265). **b** Tile plot of the expression levels of genes encoding Phase I enzymes in alphabetical order. The most up-regulated genes (medium FC > 100) are marked in *red,* and the genes with a relatively high basal expression independent of the culture conditions (medium FC < 2) are marked in *green*. See Supplementary Fig. S1 for the other gene categories (color figure online)

ALI-PBEC were generated from primary bronchial epithelial cells derived from four donors. To study the influence of culture conditions and differentiation on the transcription of genes involved in xenobiotic metabolic processes, RNA samples were collected at various time points during the establishment of the differentiated airway epithelial cultures. More specifically, RNA was isolated from sub-confluent cells grown on petri dishes under submerged conditions in KSFM medium, from cells grown to a confluent epithelial layer on tissue culture inserts in growth medium, just before (day 0) or at various days (4, 7, 11, 14, 21 and 28) after initiation of air exposure during which the cell layer gradually differentiated into mucociliary epithelium. Subsequently, RNA samples were used for gene expression analysis using Affymetrix U133 Plus 2.0 microarrays. We restricted our analysis to a subset of 362 genes encoding Phase I and Phase II enzymes, transporter and receptors (Table 1, Supplementary Table S1). Paired ANOVA analysis with donors as pairing variable and a fold-change threshold of ≥2 for the group medians identified the expression of 143 genes (BH-corrected *q* values ≤0.05) to be significantly altered during the process of differentiation (Table 1; Fig. 1a). The most dramatic change in gene expression was observed when cells were shifted from sub-confluent growth on plastic in KFSM medium to submerged growth on tissue culture inserts as a confluent layer of cells in B/D medium (Supplementary Fig. S1). seven days after culturing at the air–liquid interface, the expression level of most of the genes involved in biotransformation had stabilized. Comparing gene expression profiles at the starting condition (cells grown in KSFM) with that of ALI-PBEC after 28 days of air-exposed culturing, identified the Phase I enzymes ADH1C, ALDH1A1, CYP2B6, CYP2F1, CYP4B1 and CYP4X1 (Fig. 1b), and the Phase II enzyme GSTA1 (Supplementary Fig. S2) as being the most up-regulated genes (median FC > 100). Strong down-regulated genes (median FC > 10) included the transporter genes SLC7A5, SLC7A11 and SLC22A3, and the nuclear receptor FOXA2. The genes encoding CYP1B1 and CYP51A1 were most highly expressed with changes in gene expression being less than twofold between any two culture conditions.

### Correlation between expression and activity of CYP450

**Fig. 2:**
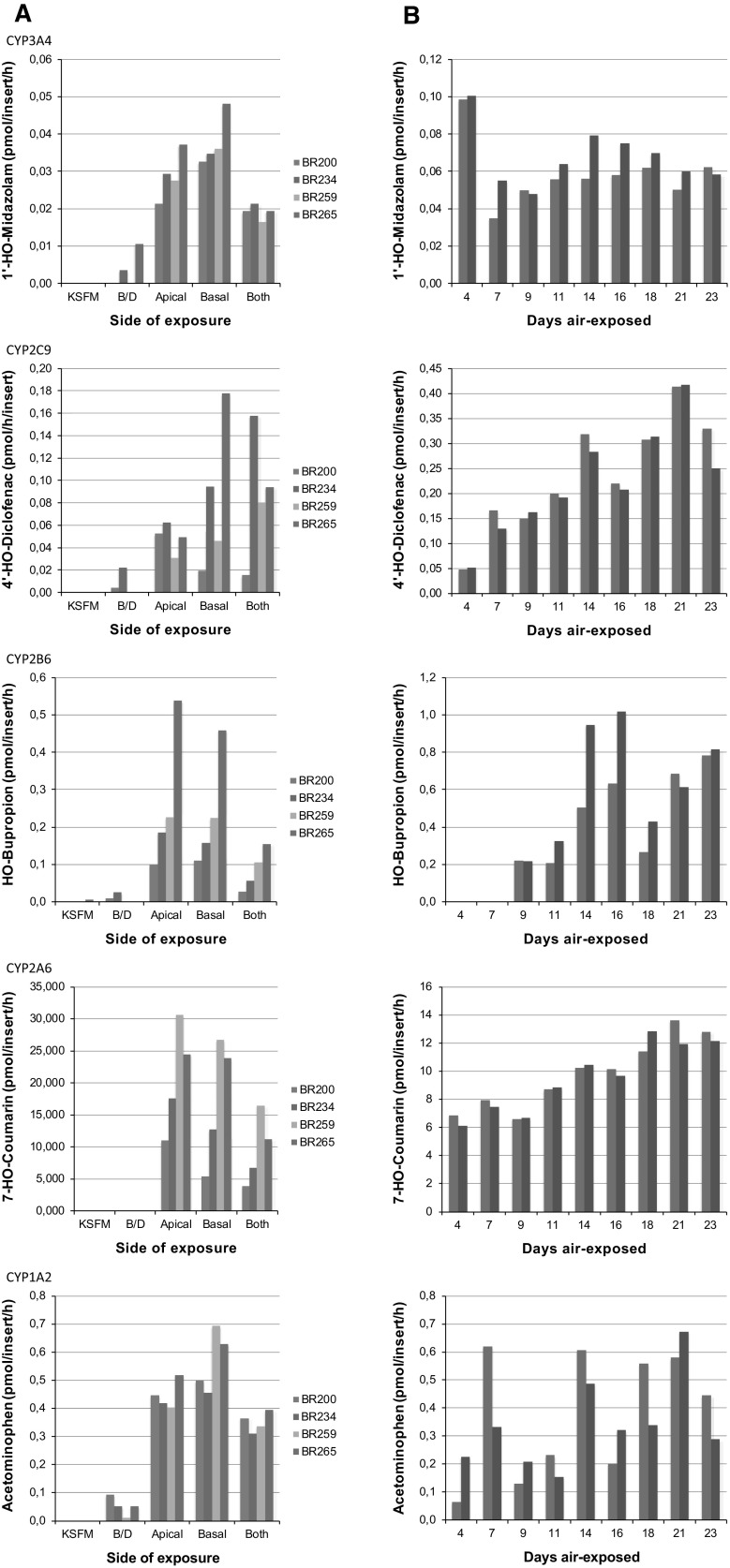
Quantification of metabolites formed after incubation of lung epithelial cells with a cocktail of substrates. **a** Comparison of metabolite formation by cells derived from four different donors grown either under submerged conditions (in KSFM or B/D medium) or after differentiation at the air–liquid interface for 14 days. The substrate cocktail was administered via the apical, basal or both sides of differentiated tissue cultures. *Each bar* represents the average metabolite level of two separate cultures. **b** Metabolite formation by cultures from donor BR265 incubated with the substrate cocktail at various days after air-exposed culturing. *Bars* represent metabolite quantification of two separate cultures

We noticed a clear enhanced expression of a large fraction of the genes involved in xenobiotic metabolism during the differentiation procedure (Fig. 1, Supplementary Fig. S2). To demonstrate that this increased expression resulted in an elevated metabolic activity, we determined the basal activity of several CYP450 s. For the first series of experiments, cells derived from four different donors were either grown under undifferentiated submerged conditions or were allowed to differentiate into ALI-PBEC for 14 days. These cultures were subsequently incubated with the substrate mixture either at the basal side, the apical side or at both sides to determine the most suitable exposure route. Metabolites present in medium samples of the various cultures were quantified for a cocktail of substrates by liquid chromatography tandem mass spectrometry. Metabolites typical for CYP1A2, CYP2A6, CYP2B6, CYP2C9 and CYP3A4 activity reached quantifiable concentrations albeit with considerable inter-donor variability (Fig. 2a). In contrast, the concentration of metabolites (mephenytoin, bufuralol and chloroxazone) typical for CYP2C19, CYP2D6 and CYP2E1 activity remained below the detection limits for all donors (data not shown). When cells were grown under submerged conditions in KSFM or B/D medium, little or no activity was observed for any of the aforementioned CYP’s. In general, the concentration of metabolites was similar for exposures via the apical and the basal side of the 3D tissue culture model. Exposure via the basal side was selected to measure metabolic activity during differentiation of tissue cultures derived from a single donor (BR265) in which metabolites indicative for CYP1A2, CYP2A6, CYP2B6, CYP2C9 and CYP3A4 activity were quantified (Fig. 2b). For most metabolites, a gradual increase in CYP activity was observed with increasing time of air-exposed culturing. The only exception was CYP3A4 for which the highest activity was measured at the earliest time point. The increase in CYP2A6 and CYP2B6 activity was paralleled by a significant increase in their gene expression, while this was not the case for CYP1A2, CYP2C9 and CYP3A4. However, the substrates used to assess activity of these CYPs, i.e., phenacetin, diclofenac and midazolam, can be metabolized in a similar fashion by, respectively, CYP2A6, CYP2C18 and CYP2B6, which all did show enhanced expression during ALI-PBEC differentiation. All together these findings demonstrate that an increased expression of genes involved in xenobiotic metabolism is accompanied by an increase, albeit somewhat delayed, in enzymatic activity.

**Fig. 3:**
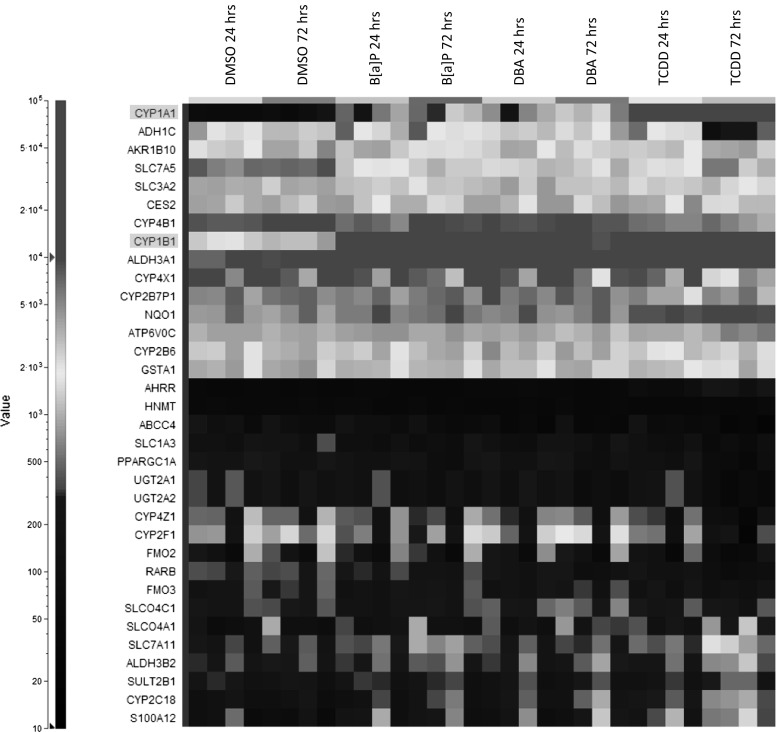
Divisive 2D hierarchical clustering with Manhattan as distance measure of differentially expressed genes (BH *q* value ≤0.05 and FC ≥ 2) upon exposure of 15 days ALI-PBEC cultures to 20 µM benzo[a]pyrene (B[a]P), 125 µM dibenzo(a,h)anthracene (DBA) or 50 nM 2,3,7,8,-tetrachlorodibenzo-para-dioxin (TCDD) for 24 or 72 h in comparison with time-matched solvent (DMSO) controls. The *four columns* per sampling time point represent the individual expression values for the four donors (from *left* to *right*; BR200, BR234, BR259 and BR265)

Although a wide variety of metabolic genes is expressed in mature ALI-PBEC cultures, we were curious how exposure to pulmonary toxicants would affect their expression. To this end, we exposed ALI-PBEC cultures to the procarcinogens benzo[a]pyrene (BaP) and dibenzo(a,h)anthracene (DBA) which are both constituents of gasoline exhaust and tobacco smoke. As a control, we investigated the transcriptional response of ALI-PBEC to TCDD, the prototypical aryl hydrocarbon receptor (AhR) ligand. Gene expression analysis of cultures 24 and 72 h after the start of exposure showed for all three compounds a strong induction of *CYP1A1* as reported previously (Baxter et al. 2015; Newland et al. 2011), while the high basal expression of *CYP1B1* was further up-regulated (Fig. 3). Consecutively, we determined CYP1A1/CYP1B1 activity using a luminogenic probe substrate (Luc-CEE) to assess if the enhanced expression of *CYP1A1* and *CYP1B1* following treatment with TCDD would lead to an increase in metabolic activity. For this purpose, we used 23-day-old ALI-PBEC cultures from donor BR234. Indeed, TCDD exposure leads to a considerable increase in metabolic activity for CYP1A1/CYP1B1 (Supplementary Fig. S3A).

As an alternative way to assess the altered metabolic activity following TCDD exposure, we exposed mature ALI-PBEC cultures, that had or had not been pretreated with TCDD, to aflatoxin B1 and determined the effect on transepithelial electrical resistance (TEER). Only cultures that had been pretreated with TCDD showed a huge impairment in barrier function in line with the ability of CYP1A1 to bioactivate aflatoxin B1 (Diaz et al. 2010) (Supplementary Fig. S3B).

### Inter-donor variation and reproducibility

**Fig. 4:**
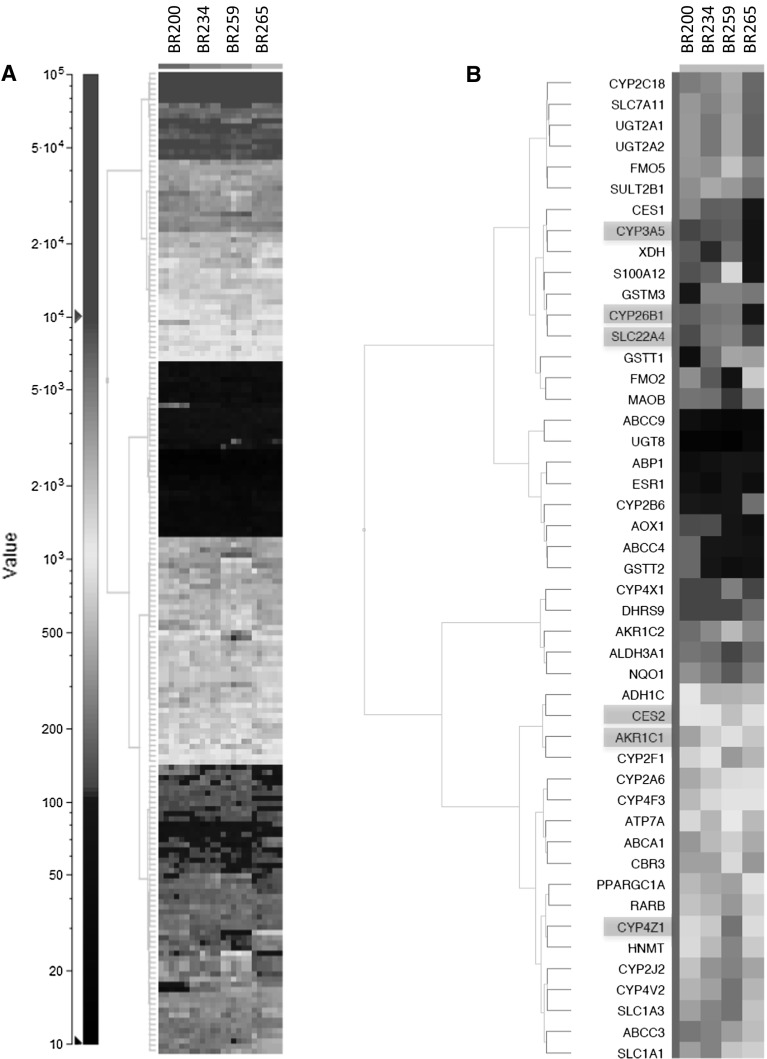
Gene expression levels of ALI-PBEC from four donors after 15 days of air-exposed culturing. **a** 2D hierarchical clustering of 190 genes with donor-dependent expression levels (BH *q* value ≤0.05). **b** 2D hierarchical clustering of the 47 genes that are differentially expressed with a FC ≥ 2 between any of the four donors. *Tiles* represent the median expression levels of six independent measurements for each donor. Genes with a FC ≥ 5 between any of the four donors are marked in *red* (color figure online)

The transcriptional changes of genes involved in xenobiotic metabolism during the differentiation procedure were in general greater than the variation in expression level between donors (Fig. 1). To get more insight into inter-donor variation in gene expression and into the reproducibility of ALI-PBEC with regard to the expression of xenobiotic metabolism-related genes, three independent studies were performed. In each study, two tissue cultures of each donor were collected for microarray analysis after 15 days of air-exposed culturing. Samples were collected 24 h after the last medium change. Applying a linear model on RMA condensed data with donor as pairing variable and study as confounding factor revealed 276 differentially expressed (*q* value ≤0.05) transcripts corresponding to 190 genes. Unsupervised hierarchical clustering of these genes separated the four donors from one another, while within the donors duplicates of each study clustered together with the exception of BR259 study 2 (Supplementary Fig. S4). A heatmap displaying the expression values of individual genes (Fig. 4a) shows that gene expression levels were highly reproducible in successive studies. Although many genes are differentially expressed between donors, the differences in fold changes are generally low. Only 47 of the differentially expressed genes have a FC ≥ 2 between the median values of any of the 4 donors (Fig. 4b), and only 6 genes display a FC ≥ 5. These genes encode the Phase I enzymes AKR1C1, CYP2F1, CYP2Z1 and FMO2, the Phase II enzyme GSTT1 and the calcium binding protein S100A12. From the 47 genes with donor-specific expression levels (FC ≥ 2), 42 (89.4 %) were also among the 143 genes that showed differential expression during the differentiation procedure. A remarkable exception is the GSTT1 gene that displayed the highest expression difference between donors (16.4-fold between donor BR200 and donor BR259) while its expression was not influenced by culture conditions or differentiation.

### Comparison with in vivo data

**Fig. 5:**
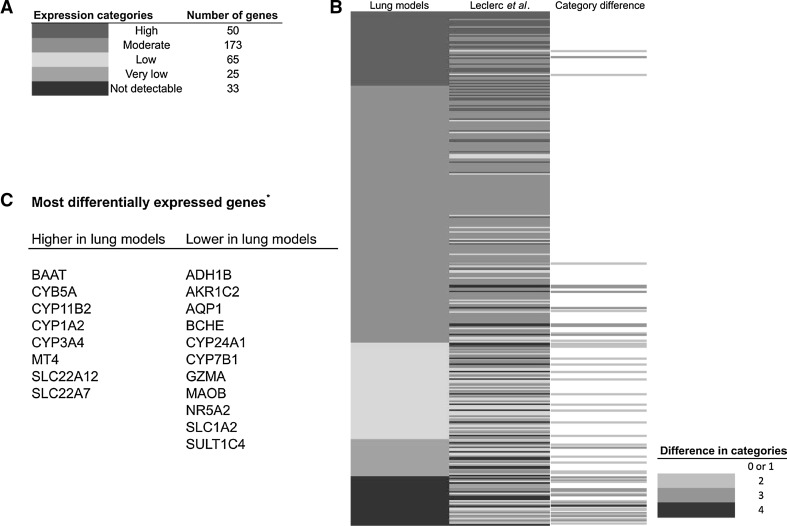
Comparison of the relative expression levels of 346 genes involved in biotransformation between ALI-PBEC cultures and macroscopically healthy bronchial tissue obtained after lung resection of lung cancer patients (data from LeClerc et al. (2011)). **a** Median gene expression levels of ALI-PBEC cultures were subdivided into five categories (Leclerc et al. 2011) ranging from not detectable to highly expressed. **b** Heatmap displaying attributed category for each gene according to our study and LeClerc et al. (2011). The extent of difference in attributed category between the two studies is indicated with *different shades* of *gray*. **c** Most differentially expressed genes between the two studies with difference in expression categories >2

So far we have demonstrated that in vitro differentiation of primary human bronchial epithelial cells leads to a reproducible and largely donor-independent increase in the transcription of genes involved in xenobiotic metabolism. Next, we compared our results with the data obtained by LeClerc et al. (2011) on xenobiotic metabolism in the lung, including bronchial mucosa. In this study, quantitative real-time PCR was used for transcriptional profiling of 380 genes involved in biotransformation from which 346 (91 %) are also present in our analyses. Based on arbitrary cutoffs for the median mRNA levels, each gene was categorized as being high, moderate, low, very low and non-detectable expressed (Fig. 5a). In order to compare the two data sets, the median expression levels of the 4 donor cultures were ranked and categorized according to the criteria used by LeClerc et al. (2011). The results are presented in Fig. 5b. From the 346 considered genes, the vast majority 295 (85.3 %) fell within the same or flanking category (Fig. 5c) indicating that gene activity of genes involved in biotransformation in ALI-PBEC to a large extent mimic the in vivo situation in the human lung.

## Discussion

Despite the fact that inhalation is one of the major exposure routes of our body to potentially hazardous substances, suitable cell systems to identify harmful effects of airborne chemicals have long been lacking (Bakand and Hayes 2010). However, currently procedures are available that allow generation of differentiated ALI-PBEC. ALI-PBEC can be brought into continuous dynamic contact with chemicals in a test atmosphere simulating in vivo inhalation exposure. Gene expression studies have shown that these 3D respiratory epithelial cultures closely resemble in vivo airway epithelia (Dvorak et al. 2011; Pezzulo et al. 2011), while also the enzymatic activity of various CYPs such as CYP1A1/1B1 and CYP2A6/2A13 is conserved (Baxter et al. 2015; Newland et al. 2011). In the present study, we determined the reproducibility and inter-individual variation of changes in gene activity of genes involved in metabolism during the differentiation of primary human bronchial epithelial cells into mature ALI-PBEC. In line with previous observations (Courcot et al. 2012), expression levels of genes involved in biotransformation were generally low when bronchial epithelial cells were cultured submerged. However, expression of many these genes was strongly elevated when cells were grown confluent on a tissue culture insert and maintained at the air–liquid interface. After about a week of air-exposed culturing, the expression levels of genes involved in biotransformation stabilized. Gene expression changes were highly reproducible among the six biological replicates for each donor, while differences in fold changes between the four donors were generally low. Only six genes displayed a FC ≥ 5 between the median values of any of the four donors (Fig. 4b).

Various CYPs are highly expressed in ALI-PBEC. CYP2B6, CYP2F1, CYP4B1 and CYP4X1 (Fig. 1b) were consistently strongly up-regulated in ALI-PBEC (median FC > 100), while in contrast CYP1B1 and CYP51A1 were already expressed at high levels in undifferentiated lung epithelial cells and only marginally changed expression during differentiation. Especially, the highly elevated expression of cytochrome P450s CYP2F1 and CYP4B1 in ALI-PBEC is of relevance since expression of these CYPs is predominantly confined to lung tissues with little or no expression in other tissues (Pavek and Dvorak 2008; Tournel et al. 2007). CYP2F1 is involved in the metabolism of various prototypical lung toxicants with potential carcinogenic effects such as naphthalene, styrene and 3-methylindole (Carr et al. 2003). One of the known substrates of the bioactivating enzyme CYP4B1 is 4-ipomeanol, a naturally occurring pulmonary protoxin present on moldy sweet potatoes (Baer et al. 2005; Choudhary et al. 2005; Parkinson et al. 2013) that can give rise to interstitial pneumonia in cattle. However, human CYP4B1 has been shown to be incapable of metabolizing IPO and its physiological function remains elusive (Wiek et al. 2015). The other highly expressed CYP4 family member CYP4X1 is a so-called orphan cytochrome P450 without an assigned biological function but also this enzyme is predominantly expressed in human trachea and aorta (Savas et al. 2005). Although expression of CYP2B6 is not restricted to pulmonary tissue, it probably plays an important role in cigarette smoke-related lung carcinogenesis. CYP2B6 metabolically activates tobacco-specific nitrosamines, including the procarcinogen 4-(methylnitrosamino)-1-(-3-pyridyl)-1-butanone (NNK), while genetic variations in CYP2B6 have been suggested as a lung cancer risk factor in smokers (Wassenaar et al. 2013). CYP1B1 is one of the main extra-hepatic cytochromes and is involved in the metabolism of multiple molecules including estradiol, retinol, tamoxifen and melatonin (Preissner et al. 2010). In humans, it is expressed in many tissues including the lung. Transcriptional activity of CYP1B1 is like CYP1A1 controlled by the aryl hydrocarbon receptor (AhR). Two other CYPs associated with lung cancer risk due to cigarette smoking, i.e., CYP2A6 and CYP2A13, displayed moderate up-regulation (FC of 9.5 and 8.9, respectively) in differentiated ALI-PBEC.

The two other identified most highly up-regulated Phase 1 enzymes ADH1C and ALDH1A1 are expressed in most human tissues including the lung and are involved in metabolic processing of acetaldehyde during alcohol metabolism. Polymorphisms of ADH1C are associated with the risk of upper aerodigestive tract cancer (Oze et al. 2009), while ALDH1A1 expression has been found to be down-regulated in various types of lung cancer (Okudela et al. 2013). The gene encoding the Phase II enzyme GSTA1 was also strongly up-regulated in ALI-PBEC (Supplementary Fig. S2) in line with the reported high expression levels in lung tissue (Leclerc et al. 2011), while its expression was found to be low or non-detectable in primary lung cells and lung cell lines, respectively (Courcot et al. 2012). Strongly reduced expression levels (FC > 10) in ALI-PBEC were found for genes encoding the solute carrier group of membrane transport proteins SLC7A5, SLC7A11 and SLC22A3. The observed low expression of these family 7 solute carriers might be related to the differentiated status of ALI-PBEC as (Courcot et al. 2012) observed higher expression of these genes in lung-derived cell lines and primary cells than in in vivo lung tissues.

Exposure of mature ALI-PBEC cultures to TCDD, the prototypical AhR ligand, and to benzo[a]pyrene and dibenz(a,h)anthracene, two aromatic hydrocarbons that are both components of tobacco smoke and diesel exhaust, resulted in a marked induction of CYP1A1. While the induction of CYP1B1 was much more moderate due to its already high basal expression in lung epithelial cells, its expression level in ALI-PBEC exceeded that of CYP1A1. In relation to their ability to metabolize polycyclic aromatic hydrocarbons (PAHs), it is important to note that in mice CYP1B1 was shown to be the dominant enzyme in metabolizing one of the most investigated PAHs, 7,12-dimethylbenz(a)anthracene (DMBA), to its carcinogenic metabolites (Buters et al. 2003).

In order to demonstrate that the increase in expression of cytochrome P450 enzymes resulted in increased metabolic activity, both submerged cultured primary cells and differentiated ALI-PBEC were incubated with a cocktail of substrates. Substrates in this cocktail were not selected to specifically measure the activity of lung typical P450 enzymes but were rather based on the hepatic cytochrome P450 system. Nevertheless, even with this suboptimal system we could demonstrate that differentiated ALI-PBEC displayed substantial metabolic activity. Although the largest increase in gene expression occurred during the transition of exponentially growing cells to a confluent layer of cells before air exposure, the enzymatic activity showed a more gradual increase with time of air-exposed culturing indicating that additional changes are required for increased gene expression of CYP genes to result in enhanced enzymatic activity. CYP activity showed the largest increase when after several days of air-exposed culturing differentiated cells, such as ciliated, goblet and club cells, appeared in the cultures. Especially, the latter cell type is reported to possess metabolic capacity. In mice for example, CYP2F2 is primarily expressed in the lung by club cells (Ritter et al. 1991) and exposure to the CYP2F2 substrate styrene results in club cell toxicity, increased proliferation of lung cells and ultimately in the formation of lung tumors. In contrast, rats express much lower levels of CYP2F2 and do not develop lung tumors upon styrene exposure (Cruzan et al. 2012, 2013). Expression in humans is restricted to the CYP2F1 isoform that has a lower catalytic activity than CYP2F2 in styrene metabolism, but which displayed a strong up-regulation in differentiated ALI-PBEC. The ability to recognize various cell types in ALI-PBEC combined with single-cell analysis of potential mutagenic activities (e.g., induction of DNA breaks by gamma-H2AX foci formation) of certain metabolites will enable identification of the cell types responsible for the metabolic activation of and sensitivity for airborne protoxicants and procarcinogens in humans.

Altogether, our data indicate that differentiated ALI-PBEC mimic to a large extent in vivo epithelium in terms of expression and activity of genes involved in xenobiotic and drug metabolic processes. An essential step for their application as test vehicles for risk assessment of airborne substances is the development of assays able to quantify induced (geno)toxicity within the models. With such assays in place, ALI-PBEC might aid in elucidating discrepancies in sensitivity between rodents and humans for certain inhaled compounds. In addition, these models might be used to investigate the effects of various polymorphisms in for instance Phase I enzymes that are present in the human population on the biotransforming capacities of human lung tissue. Other opportunities for ALI-PBEC include the possibility to investigate the effect of (geno)toxic exposures on the differentiation process, the use of patient-specific ALI-PBEC and studies on the effect of pro-inflammatory stimuli.

Obviously further validation is required using ALI-PBEC from larger numbers of well-characterized donors, but it is clear that differentiated ALI-PBEC are a useful model to investigate the cellular effects of airborne chemicals and have the potential to reduce and eventually replace inhalation studies that are currently performed with rodents.

## Electronic supplementary material

Below is the link to the electronic supplementary material.

Supplementary material 1 (DOCX 11 kb)

Supplementary material 2 (PDF 194 kb)

Supplementary material 3 (PDF 13 kb)
