# Supplementary material for: Xenobiotic metabolism in differentiated human bronchial epithelial cells
Source: Arch Toxicol. 2016 Oct 13;91(5):2093–105. doi: 10.1007/s00204-016-1868-7 (PMC5399058; doi:10.1007/s00204-016-1868-7)
Supplement: Supplementary file 1 — Supplementary material 1 (DOCX 11 kb) [file 204_2016_1868_MOESM1_ESM.docx]

**Supplementary Data Description**

**Table S1** List of considered genes involved in xenobiotic and drug metabolism together with their representative probe sets.

**Fig. S1** Tile plot displaying in alphabetical order the expression levels of genes encoding phase II enzymes, receptors, transporter proteins and other genes. Strong up- (median FC>100) and down-regulated genes (median FC>10) are marked in red.

**Fig. S2** Numbers of differentially expressed genes between subsequent sampling time points.

**Fig. S3** (A) Induction of CYP1A1/CYP1B1 activity after TCDD exposure. CYP1A1/CYP1B1 activity was measured using the P450-Glo assay in ALI-PBEC cultures of donor BR234 after 72h exposure to 100 nM TCDD and compared with the solvent controls (B) Increased toxicity of AFB1) in ALI-PBEC cultures after pre-treatment with TCDD. ALI-PBEC cultures were exposed for 24h to 2 µM AFB1 with and without 72h pre-treatment with 100 nM TCDD. Toxicity was accessed by the measurement of trans-epithelial electrical resistance (TEER) of the same ALI-PBEC cultures after TCDD pre-treatment and after AFB1 exposure. Solvent (DMSO) controls were included for all conditions. Without TCDD pre-treatment AFB1 exposure resulted in only a minor reduction in TEER levels whereas after TCDD pre-treatment hardly any TEER was remaining

**Fig. S4** Gene expression levels of ALI-PBEC cultures from four donors after 15 days of air-exposed culturing. Unsupervised hierarchical clustering of 276 differentially expressed transcripts. A total of six samples per donor (BR200, BR234, BR259 and BR265) obtained from three independent experiments (depicted 1-3) each with two biological replicates were analysed.
