# Supplementary figures and images for: Xenobiotic metabolism in differentiated human bronchial epithelial cells
Source: Arch Toxicol. 2016 Oct 13;91(5):2093–105. doi: 10.1007/s00204-016-1868-7 (PMC5399058; doi:10.1007/s00204-016-1868-7)

Supplementary Fig. S1

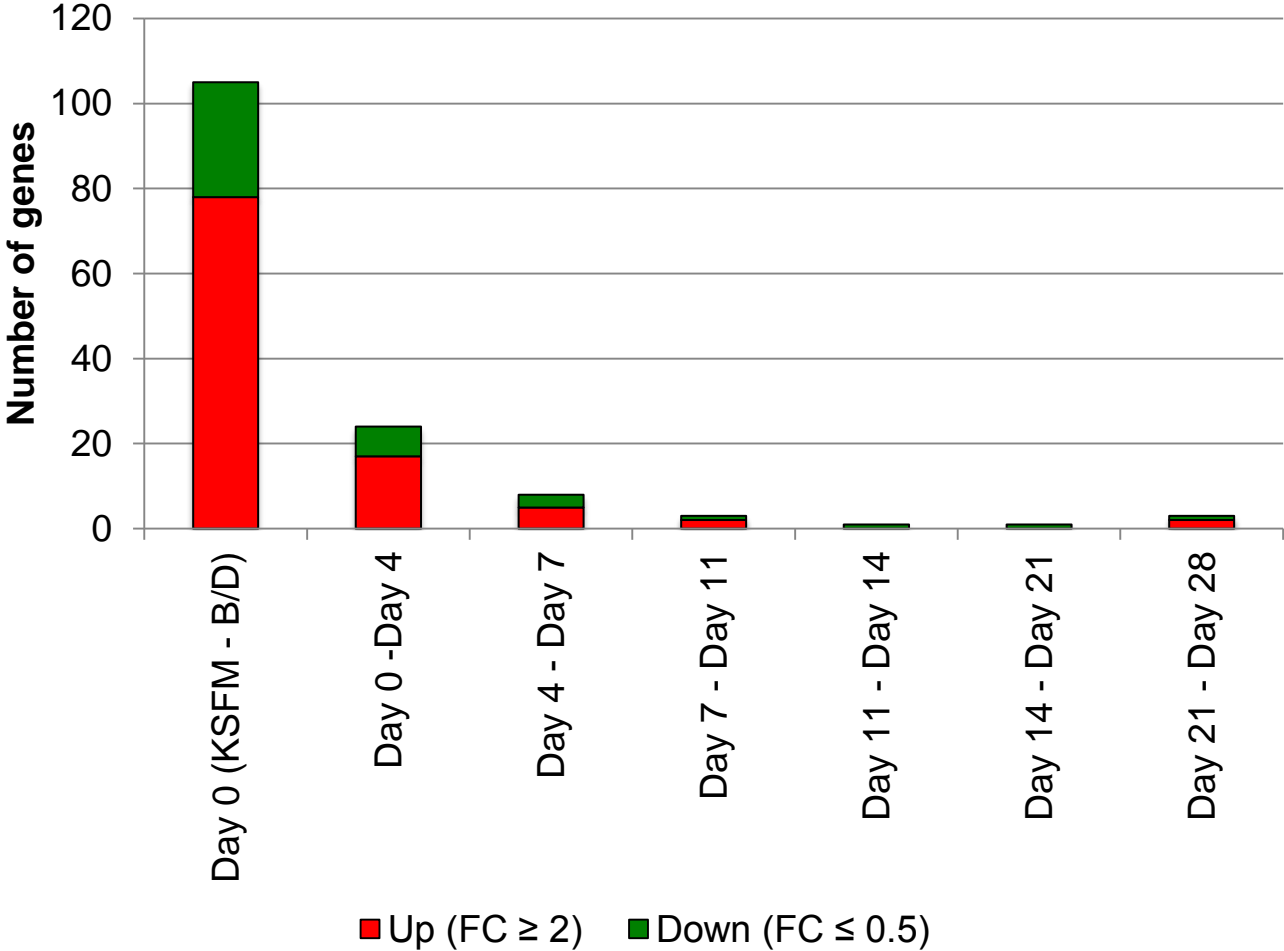

Supplementary Fig. S2

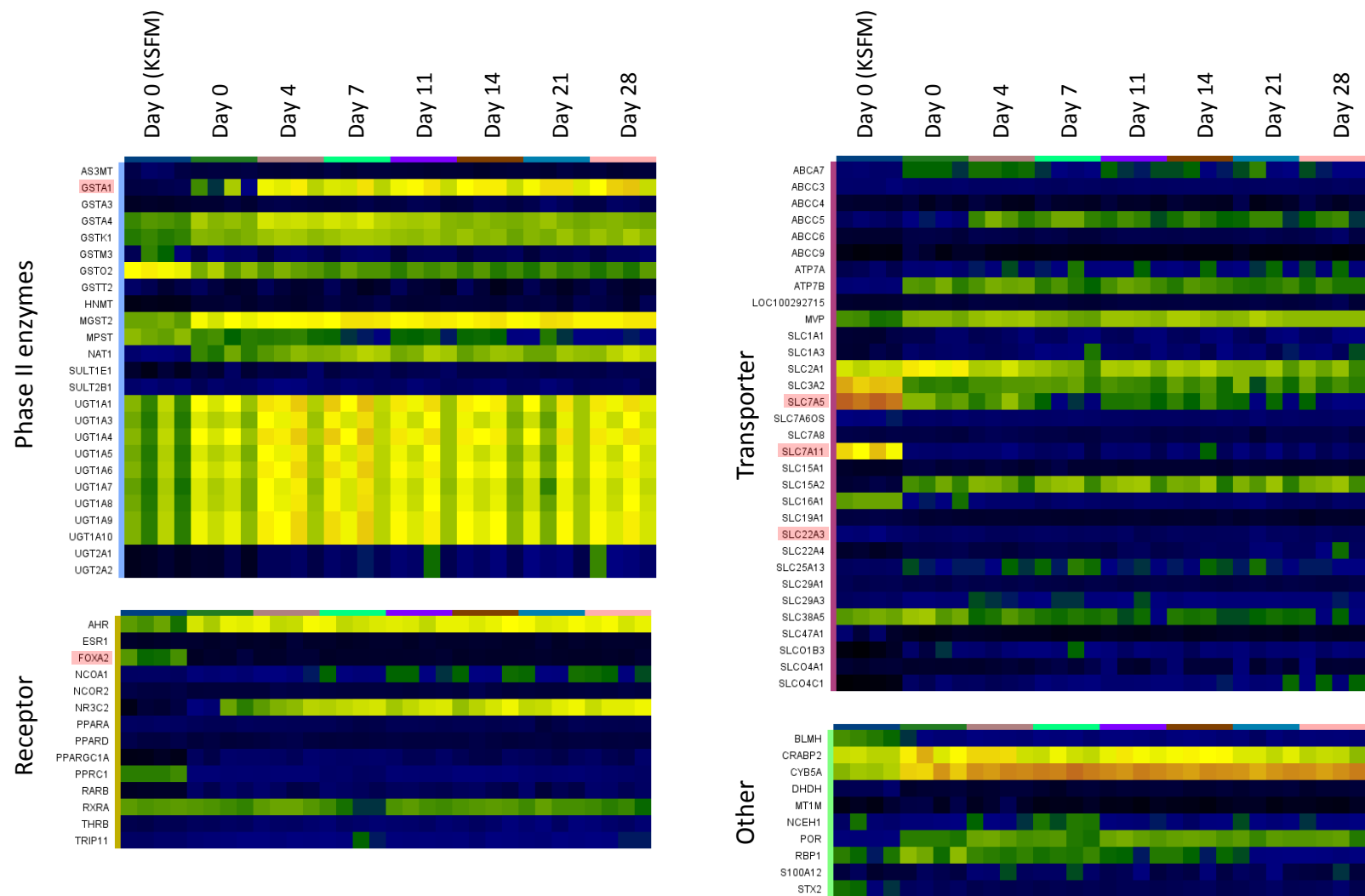

Supplementary Fig. S3

A

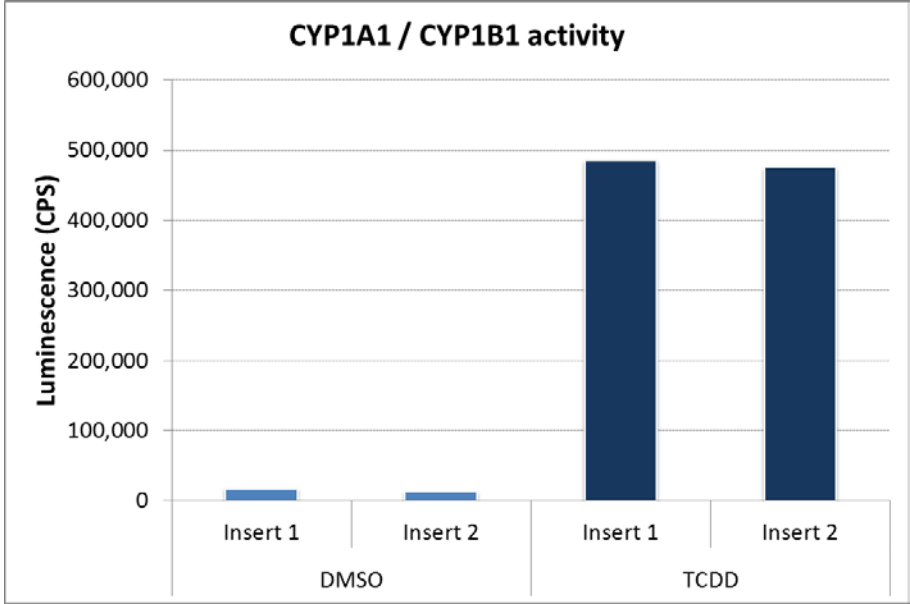

B

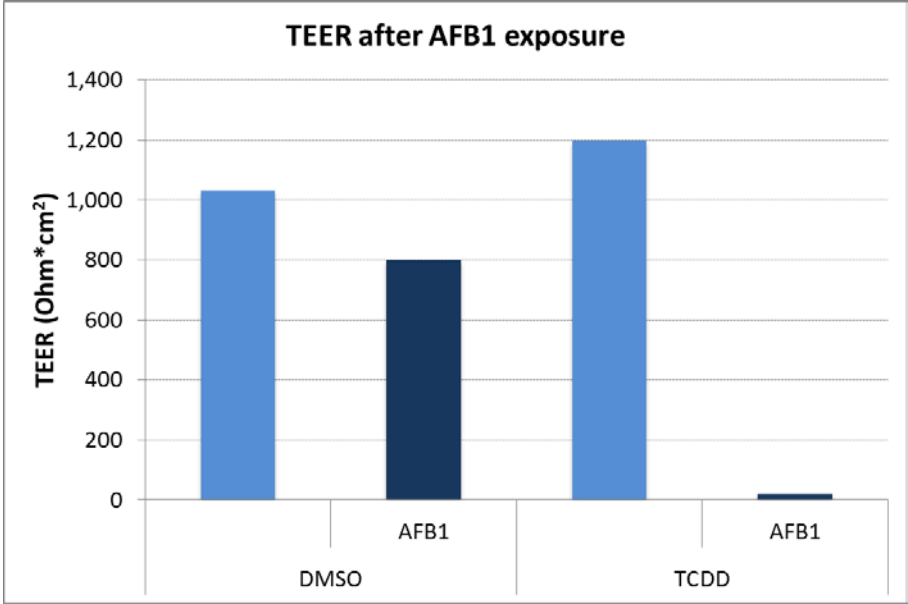

Supplementary Fig. S4

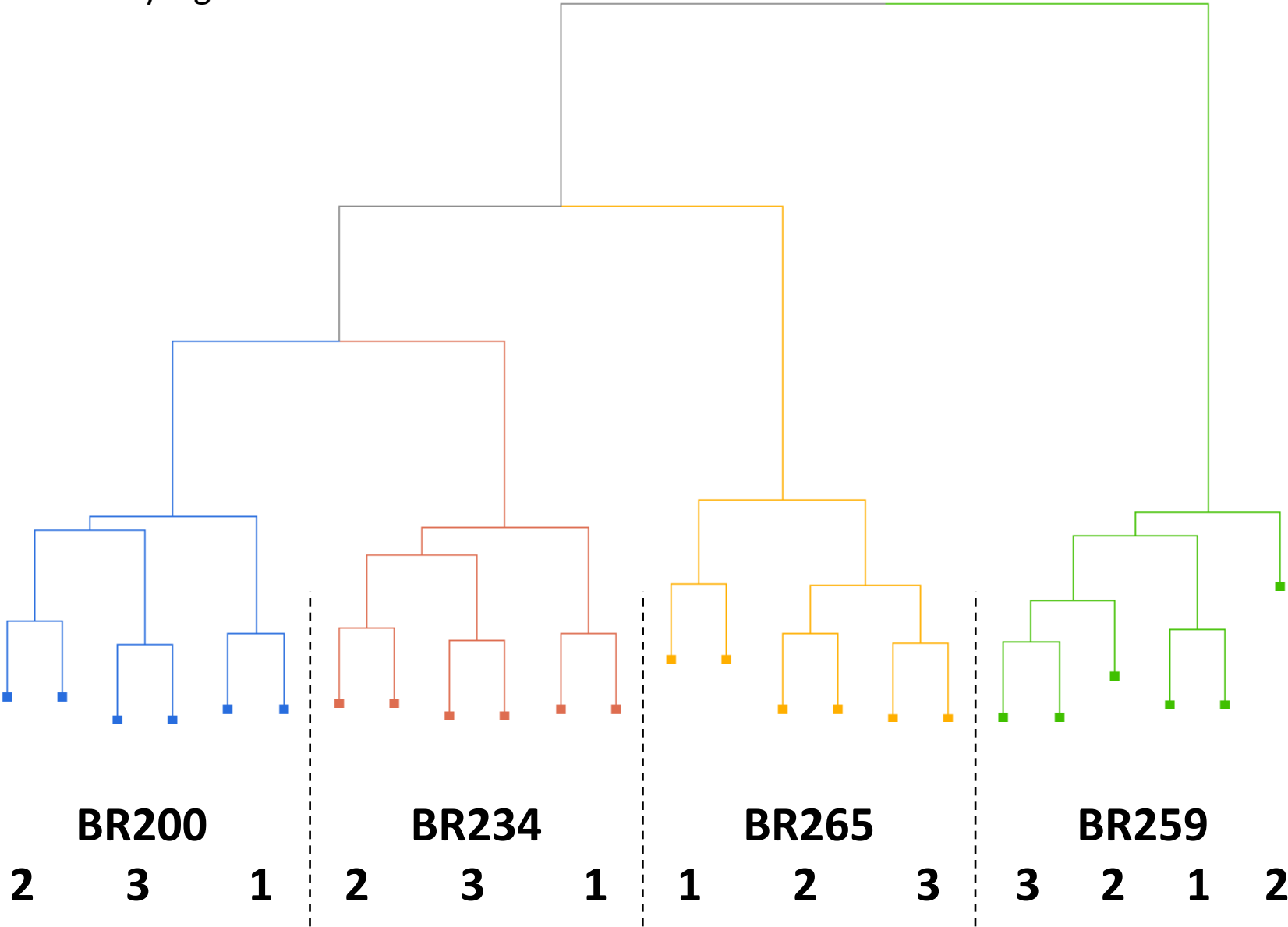

Supplement: Supplementary file 2 — Supplementary material 2 (PDF 194 kb) [file 204_2016_1868_MOESM2_ESM.pdf]
