# Supplementary material for: Xenobiotic metabolism in differentiated human bronchial epithelial cells
Source: Arch Toxicol. 2016 Oct 13;91(5):2093–105. doi: 10.1007/s00204-016-1868-7 (PMC5399058; doi:10.1007/s00204-016-1868-7)
Supplement: Supplementary file 3 — Supplementary material 3 (PDF 13 kb) [file 204_2016_1868_MOESM3_ESM.pdf]

Table S1. List of considered genes involved in xenobiotic and drug metabolism together with their representative probe sets.

**Category**	**Gene Symbol** (Affymetrix IVT)	**Probe sets** (present on the Affymetrix Human Genome U133 Plus 2.0 GeneChip array:				
Phase I enzymes	AADAC	205969_at				
Phase I enzymes	ABP1	203559_s_at				
Phase I enzymes	ADH1A	207820_at				
Phase I enzymes	ADH1B	209612_s_at	209613_s_at	209614_at	243544_at	
Phase I enzymes	ADH1C	206262_at				
Phase I enzymes	ADH4	231675_s_at	231678_s_at	231703_s_at		
Phase I enzymes	ADH5	208847_s_at	208848_at			
Phase I enzymes	ADH6	207544_s_at	214261_s_at			
Phase I enzymes	ADH7	210505_at				
Phase I enzymes	ADHFE1	227113_at				
Phase I enzymes	AKR1A1	201900_s_at	241978_at			
Phase I enzymes	AKR1B1	201272_at				
Phase I enzymes	AKR1B10	206561_s_at				
Phase I enzymes	AKR1C1	217626_at	244266_at	1562102_at		
Phase I enzymes	AKR1C1 : AKR1C2	1555854_at				
Phase I enzymes	AKR1C2	1560098_at				
Phase I enzymes	AKR1C3	209160_at				
Phase I enzymes	AKR1C4	210558_at				
Phase I enzymes	AKR1D1	207102_at				
Phase I enzymes	AKR7A2	202139_at	214259_s_at			
Phase I enzymes	ALDH16A1	227592_at				
Phase I enzymes	ALDH18A1	217791_s_at	222416_at			
Phase I enzymes	ALDH1A1	212224_at				
Phase I enzymes	ALDH1A2	207015_s_at	207016_s_at			
Phase I enzymes	ALDH1A3	203180_at				
Phase I enzymes	ALDH1B1	209645_s_at				
Phase I enzymes	ALDH1L1	205208_at	215798_at			
Phase I enzymes	ALDH2	201425_at				
Phase I enzymes	ALDH3A1	205623_at				
Phase I enzymes	ALDH3A2	202053_s_at	202054_s_at	210544_s_at		
Phase I enzymes	ALDH3B1	205640_at	211004_s_at	1565731_at		
Phase I enzymes	ALDH3B2	204941_s_at	204942_s_at			
Phase I enzymes	ALDH4A1	203722_at	211552_s_at			
Phase I enzymes	ALDH5A1	203608_at	203609_s_at			
Phase I enzymes	ALDH6A1	204289_at	204290_s_at	221589_s_at	221590_s_at	
Phase I enzymes	ALDH7A1	208950_s_at	208951_at	213591_at		
Phase I enzymes	ALDH8A1	220148_at				
Phase I enzymes	ALDH9A1	201612_at				
Phase I enzymes	AOC2	207064_s_at				
Phase I enzymes	AOC3	204894_s_at				
Phase I enzymes	AOX1	205082_s_at	205083_at			
Phase I enzymes	BCHE	205433_at				
Phase I enzymes	CBR1	209213_at				
Phase I enzymes	CBR3	205379_at				
Phase I enzymes	CBR4	213626_at	244052_at	1553693_s_at		
Phase I enzymes	CES1	209616_s_at				
Phase I enzymes	CES2	209667_at				
Phase I enzymes	CES3	234008_s_at				
Phase I enzymes	CES4	206824_at				
Phase I enzymes	CES7	1553465_a_at				
Phase I enzymes	CYP11A1	204309_at				
Phase I enzymes	CYP11B1	214610_at	1552493_s_at			
Phase I enzymes	CYP11B2	214630_at				
Phase I enzymes	CYP17A1	205502_at	1562573_at			
Phase I enzymes	CYP19A1	203475_at	240705_at	240863_at	1554296_at	1560295_at
Phase I enzymes	CYP1A1	205749_at				
Phase I enzymes	CYP1A2	207609_s_at				
Phase I enzymes	CYP1B1	202434_s_at	202435_s_at	202436_s_at	202437_s_at	
Phase I enzymes	CYP20A1	219565_at				
Phase I enzymes	CYP21A2	214622_at				
Phase I enzymes	CYP24A1	206504_at				
Phase I enzymes	CYP26A1	206424_at				
Phase I enzymes	CYP26B1	219825_at	234721_s_at			
Phase I enzymes	CYP27A1	203979_at				
Phase I enzymes	CYP27B1	205676_at				
Phase I enzymes	CYP27C1	1568868_at				
Phase I enzymes	CYP2A13	208327_at				
Phase I enzymes	CYP2A6	1494_f_at				
Phase I enzymes	CYP2B6	206755_at				
Phase I enzymes	CYP2B6 : CYP2B7P1	206754_s_at				
Phase I enzymes	CYP2C18	208126_s_at	215103_at			
Phase I enzymes	CYP2C19	216058_s_at				
Phase I enzymes	CYP2C8	208147_s_at	219903_s_at			
Phase I enzymes	CYP2C9	214419_s_at	214420_s_at	217558_at		
Phase I enzymes	CYP2D6	207498_s_at	215809_at	217468_at		
Phase I enzymes	CYP2E1	1431_at	209975_at	209976_s_at	222100_at	
Phase I enzymes	CYP2F1	207913_at				
Phase I enzymes	CYP2J2	205073_at				
Phase I enzymes	CYP2R1	207786_at	227109_at			
Phase I enzymes	CYP2S1	223385_at				
Phase I enzymes	CYP2U1	216720_at	226393_at	226402_at	1554853_at	
Phase I enzymes	CYP2W1	220562_at				
Phase I enzymes	CYP39A1	220432_s_at	244407_at	1553977_a_at		
Phase I enzymes	CYP3A4	210726_at				
Phase I enzymes	CYP3A5	205765_at	214234_s_at	214235_at	243015_at	
Phase I enzymes	CYP3A7	205939_at	243609_at			
Phase I enzymes	CYP46A1	220331_at				
Phase I enzymes	CYP4A11	1554931_at				
Phase I enzymes	CYP4A11 : CYP4A22	1554837_a_at				
Phase I enzymes	CYP4B1	210096_at	1555497_a_at			
Phase I enzymes	CYP4F11	206153_at				
Phase I enzymes	CYP4F12	206539_s_at				
Phase I enzymes	CYP4F2 : CYP4F3	206514_s_at				
Phase I enzymes	CYP4F22	244692_at				
Phase I enzymes	CYP4F3	206515_at				
Phase I enzymes	CYP4F8	210576_at				
Phase I enzymes	CYP4V2	226745_at	228391_at	235719_at		
Phase I enzymes	CYP4X1	227702_at				
Phase I enzymes	CYP4Z1	237395_at				
Phase I enzymes	CYP51A1	202314_at	216607_s_at			
Phase I enzymes	CYP7A1	207406_at				
Phase I enzymes	CYP7B1	207386_at				
Phase I enzymes	CYP8B1	232494_at				
Phase I enzymes	DHRS2	206463_s_at	214079_at			
Phase I enzymes	DHRS4 : DHRS4L2	218021_at				
Phase I enzymes	DHRS9	219799_s_at				
Phase I enzymes	DPYD	204646_at	1554534_at	1554536_at		
Phase I enzymes	EPHX1	202017_at				
Phase I enzymes	EPHX2	209368_at				
Phase I enzymes	ESD	209009_at	215096_s_at	228162_at	240808_at	
Phase I enzymes	FMO1	205666_at				
Phase I enzymes	FMO2	211726_s_at	228268_at			
Phase I enzymes	FMO3	206496_at	40665_at			
Phase I enzymes	FMO4	206263_at				
Phase I enzymes	FMO5	205776_at	215300_s_at	1569688_at		
Phase I enzymes	HSD17B10	202282_at				
Phase I enzymes	MAOA	204388_s_at	204389_at	212741_at		
Phase I enzymes	MAOB	204041_at	1561009_at	1561010_a_at		
Phase I enzymes	NQO1	201467_s_at	201468_s_at	210519_s_at		
Phase I enzymes	NQO2	203814_s_at				
Phase I enzymes	PAOX	221941_at	50400_at			
Phase I enzymes	PON1	206344_at	206345_s_at			
Phase I enzymes	PON2	201876_at	210830_s_at	242700_at		
Phase I enzymes	PON3	213695_at				
Phase I enzymes	SPR	203458_at				
Phase I enzymes	SUOX	204067_at	1553030_a_at			
Phase I enzymes	XDH	210301_at	241994_at			
Phase I enzymes	ZNF498	231704_at				
Phase II enzymes	AANAT	207225_at				
Phase II enzymes	AS3MT	223652_at				
Phase II enzymes	BAAT	206913_at				
Phase II enzymes	COMT	208817_at	208818_s_at	213981_at	216204_at	
Phase II enzymes	GGT1	233837_at				
Phase II enzymes	GLYAT	206930_at	222083_at	231683_at		
Phase II enzymes	GNMT	210328_at				
Phase II enzymes	GSTA1	203924_at	215766_at			
Phase II enzymes	GSTA3	222102_at				
Phase II enzymes	GSTA4	202967_at	235405_at			
Phase II enzymes	GSTK1	217751_at	243325_at			

Phase II enzymes	GSTM3	202554_s_at	235867_at				
Phase II enzymes	GSTM4	204149_s_at					
Phase II enzymes	GSTM5	205752_s_at					
Phase II enzymes	GSTO1	201470_at	1557915_s_at				
Phase II enzymes	GSTO2	227163_at					
Phase II enzymes	GSTP1	200824_at					
Phase II enzymes	GSTT1	203815_at	232193_at				
Phase II enzymes	GSTT2	205439_at					
Phase II enzymes	GSTZ1	209531_at					
Phase II enzymes	HNMT	204110_at	204111_at	204112_s_at	228772_at	1554303_at	
Phase II enzymes	INMT	224061_at					
Phase II enzymes	MGST1	239001_at	1565162_s_at				
Phase II enzymes	MGST2	204168_at					
Phase II enzymes	MGST3	201403_s_at	244122_at				
Phase II enzymes	MPST	203524_s_at					
Phase II enzymes	NAT1	214440_at					
Phase II enzymes	NAT2	206797_at					
Phase II enzymes	NNMT	202237_at	202238_s_at	231559_at			
Phase II enzymes	PNMT	206793_at					
Phase II enzymes	SULT1A1	238995_at					
Phase II enzymes	SULT1A3 : SULT1A4	222094_at					
Phase II enzymes	SULT1B1	207601_at					
Phase II enzymes	SULT1C2	205342_s_at	205343_at	211470_s_at	240200_at		
Phase II enzymes	SULT1C4	1553321_a_at					
Phase II enzymes	SULT1E1	219934_s_at	222940_at				
Phase II enzymes	SULT2A1	206292_s_at	206293_at				
Phase II enzymes	SULT2B1	205759_s_at					
Phase II enzymes	SULT4A1	219425_at					
Phase II enzymes	TPMT	203671_at	238272_at				
Phase II enzymes	TST	209605_at					
Phase II enzymes	UGT1A	232655_at					
Phase II enzymes	UGT1A1 : UGT1A10 : UGT1A3 : UGT1A4 : UGT1A5 : UGT1A6 : UGT1A7 : UGT1A8 : UGT1A9	208596_s_at	215125_s_at				
Phase II enzymes	UGT1A10 : UGT1A7 : UGT1A8	221304_at					
Phase II enzymes	UGT1A6	232654_s_at					
Phase II enzymes	UGT1A8 : UGT1A9	221305_s_at					
Phase II enzymes	UGT2A1 : UGT2A2	207958_at					
Phase II enzymes	UGT2B15	217175_at					
Phase II enzymes	UGT2B17	207245_at					
Phase II enzymes	UGT2B4	206505_at					
Phase II enzymes	UGT3A1	235904_at	236597_at	237572_at			
Phase II enzymes	UGT8	208358_s_at	228956_at				
Transporter genes	ABCA1	203504_s_at	203505_at	216066_at	1570279_at		
Transporter genes	ABCA2	210099_at	210100_s_at	212772_s_at			
Transporter genes	ABCA3	204343_at					
Transporter genes	ABCA4	210082_at	1569102_at				
Transporter genes	ABCA7	219577_s_at					
Transporter genes	ABCA8	204719_at	1565778_at	1565780_at			
Transporter genes	ABCB1	209993_at	243951_at				
Transporter genes	ABCB1 : ABCB4	209994_s_at					
Transporter genes	ABCB10	223320_s_at					
Transporter genes	ABCB11	208288_at	211224_s_at				
Transporter genes	ABCB4	207819_s_at	1570505_at				
Transporter genes	ABCB5	240717_at	243167_at	1555371_at	1569072_s_at		
Transporter genes	ABCB6	203191_at	203192_at				
Transporter genes	ABCB7	209620_s_at					
Transporter genes	ABCB8	206317_s_at	229539_at				
Transporter genes	ABCB9	207321_s_at	214209_s_at	1555323_at			
Transporter genes	ABCC1	202804_at	202805_s_at				
Transporter genes	ABCC10	213485_s_at					
Transporter genes	ABCC11	224146_s_at	1554911_at				
Transporter genes	ABCC12	1552590_a_at	1553410_a_at				
Transporter genes	ABCC2	206155_at					
Transporter genes	ABCC3	208161_s_at	209641_s_at	214979_at	242553_at		
Transporter genes	ABCC4	203196_at	243928_s_at	1554918_a_at	1555039_a_at		
Transporter genes	ABCC5	209380_s_at	226363_at	1558460_at			
Transporter genes	ABCC6	214033_at					
Transporter genes	ABCC6 : LOC100292715	208480_s_at	215559_at				
Transporter genes	ABCC8	210245_at	210246_s_at				
Transporter genes	ABCC9	208462_s_at	208561_at	208562_s_at	235578_at	1557374_at	
Transporter genes	ABCD4	203981_s_at	203982_s_at				
Transporter genes	ABCG2	209735_at					
Transporter genes	ABCG8	231751_at					
Transporter genes	AQP1	207542_s_at	209047_at				
Transporter genes	AQP7	206955_at					
Transporter genes	AQP9	205568_at					
Transporter genes	ATP6V0C	200954_at	36994_at				
Transporter genes	ATP7A	205197_s_at	205198_s_at				
Transporter genes	ATP7B	204624_at					
Transporter genes	KCNK9	224072_s_at	238870_at				
Transporter genes	MVP	202180_s_at					
Transporter genes	SLC10A1	207185_at					
Transporter genes	SLC10A2	207095_at					
Transporter genes	SLC15A1	207254_at	211349_at				
Transporter genes	SLC15A2	205316_at	205317_s_at	240159_at			
Transporter genes	SLC16A1	202234_s_at	202235_at	202236_s_at	209900_s_at	1557918_s_at	
Transporter genes	SLC18A2	205857_at	1553328_a_at				
Transporter genes	SLC19A1	209776_s_at	209777_s_at	211576_s_at	229639_s_at	1555952_at	1555953_at
Transporter genes	SLC19A2	209681_at					
Transporter genes	SLC19A3	220736_at	239345_at				
Transporter genes	SLC1A1	206396_at	213664_at				
Transporter genes	SLC1A2	208389_s_at	225491_at	1558009_at	1558010_s_at		
Transporter genes	SLC1A3	202800_at	1569054_at				
Transporter genes	SLC1A6	206882_at	1554592_a_at	1554593_s_at			
Transporter genes	SLC1A7	207355_at	210923_at	243623_at			
Transporter genes	SLC22A1	207201_s_at					
Transporter genes	SLC22A11	220100_at					
Transporter genes	SLC22A12	237799_at					
Transporter genes	SLC22A16	232232_s_at	232233_at				
Transporter genes	SLC22A2	207429_at					
Transporter genes	SLC22A3	205421_at					
Transporter genes	SLC22A4	205896_at					
Transporter genes	SLC22A5	205074_at					
Transporter genes	SLC22A6	210343_s_at					
Transporter genes	SLC22A7	220554_at	221661_at	221662_s_at	231398_at	1555553_a_at	
Transporter genes	SLC22A8	221298_s_at	231352_at				
Transporter genes	SLC22A9	231625_at					
Transporter genes	SLC25A13	203775_at	229061_s_at	229081_at			
Transporter genes	SLC28A1	207560_at	231187_at				
Transporter genes	SLC28A2	207249_s_at	216432_at				
Transporter genes	SLC28A3	220475_at					
Transporter genes	SLC29A1	201801_s_at	201802_at				
Transporter genes	SLC29A2	204717_s_at	1553540_a_at	1560062_at	1560149_at		
Transporter genes	SLC29A3	219344_at					
Transporter genes	SLC29A4	227281_at					
Transporter genes	SLC2A1	201249_at	201250_s_at				
Transporter genes	SLC31A1	203971_at	235013_at	236217_at			
Transporter genes	SLC38A1	218237_s_at	224579_at	224580_at	236857_at		
Transporter genes	SLC38A2	220924_s_at					
Transporter genes	SLC38A5	234973_at					
Transporter genes	SLC3A1	205799_s_at	239667_at				
Transporter genes	SLC3A2	200924_s_at					
Transporter genes	SLC47A1	219525_at					
Transporter genes	SLC47A2	231068_at					
Transporter genes	SLC5A4	215960_at					
Transporter genes	SLC6A3	206836_at					
Transporter genes	SLC6A4	207519_at	242009_at				
Transporter genes	SLC7A11	207528_s_at	209921_at	217678_at			
Transporter genes	SLC7A5	201195_s_at					
Transporter genes	SLC7A6	203578_s_at	203579_s_at	203580_s_at			
Transporter genes	SLC7A6OS	229153_at	232057_at				
Transporter genes	SLC7A7	204588_s_at					
Transporter genes	SLC7A8	216092_s_at	216603_at	216604_s_at	217248_s_at		
Transporter genes	SLCO1A2	207308_at	211480_s_at	211481_at			
Transporter genes	SLCO1B1	210366_at					
Transporter genes	SLCO1B3	206354_at					
Transporter genes	SLCO1C1	220460_at					
Transporter genes	SLCO2A1	204368_at					
Transporter genes	SLCO2B1	203472_s_at	203473_at				
Transporter genes	SLCO3A1	210542_s_at	219229_at	227367_at	229776_at		
Transporter genes	SLCO4A1	219911_s_at					
Transporter genes	SLCO4C1	207596_at	222071_s_at				
Transporter genes	SLCO5A1	220984_s_at					

Transporter genes	SLCO6A1	1552745_at							
Transporter genes	VDAC2	211662_s_at							
Transporter genes	VDAC3	208844_at	208845_at	208846_s_at					
Nuclear receptors & Trancription factors	AHR	202820_at							
Nuclear receptors & Trancription factors	AHRR	229354_at							
Nuclear receptors & Trancription factors	AIP	201781_s_at	201782_s_at						
Nuclear receptors & Trancription factors	ARNT	210828_s_at	218221_at	230619_at	231016_s_at	233724_at			
Nuclear receptors & Trancription factors	ARNT2	202986_at							
Nuclear receptors & Trancription factors	CREBBP	202160_at	211808_s_at	228177_at	235858_at				
Nuclear receptors & Trancription factors	EP300	202221_s_at	213579_s_at						
Nuclear receptors & Trancription factors	ESR1	205225_at	211235_s_at	215552_s_at	217163_at				
Nuclear receptors & Trancription factors	ESR2	210780_at	211119_at	1569554_at					
Nuclear receptors & Trancription factors	FOXA2	210103_s_at	214312_at	40284_at					
Nuclear receptors & Trancription factors	FOXO1	202723_s_at	202724_s_at	228484_s_at					
Nuclear receptors & Trancription factors	HIF1A	200989_at							
Nuclear receptors & Trancription factors	HIF3A	219319_at	222123_s_at	222124_at	232669_at	233517_s_at	1555318_at	1556069_s_at	
Nuclear receptors & Trancription factors	HNF4A	214832_at	214851_at	216889_s_at	230914_at				
Nuclear receptors & Trancription factors	HSP90AA1	210211_s_at	211968_s_at	211969_at	214328_s_at				
Nuclear receptors & Trancription factors	KEAP1	202417_at							
Nuclear receptors & Trancription factors	NCOA1	209105_at	209106_at	210249_s_at	231289_at				
Nuclear receptors & Trancription factors	NCOA2	205731_s_at	205732_s_at	215605_at					
Nuclear receptors & Trancription factors	NCOA3	207700_s_at	209061_at	211352_s_at	1562439_at				
Nuclear receptors & Trancription factors	NCOR1	200854_at	200857_s_at	234313_at					
Nuclear receptors & Trancription factors	NCOR2	207760_s_at	208888_s_at	208889_s_at	236025_at				
Nuclear receptors & Trancription factors	NFE2L2	201146_at	1567013_at	1567014_s_at	1567015_at				
Nuclear receptors & Trancription factors	NR0B2	206410_at							
Nuclear receptors & Trancription factors	NR1H2	218215_s_at							
Nuclear receptors & Trancription factors	NR1H3	203920_at							
Nuclear receptors & Trancription factors	NR1H4	206340_at	243800_at	1554375_a_at					
Nuclear receptors & Trancription factors	NR1I2	207202_s_at	207203_s_at						
Nuclear receptors & Trancription factors	NR1I3	207007_at	1570188_at						
Nuclear receptors & Trancription factors	NR3C1	201866_s_at	211671_s_at	216321_s_at					
Nuclear receptors & Trancription factors	NR3C2	205259_at							
Nuclear receptors & Trancription factors	NR5A2	208337_s_at	208343_s_at	210174_at	1560469_at				
Nuclear receptors & Trancription factors	PPARA	206870_at	210771_at	223437_at	223438_s_at	226978_at	244689_at	1558631_at	1560981_a_at
Nuclear receptors & Trancription factors	PPARD	208044_s_at	210636_at	37152_at	242218_at				
Nuclear receptors & Trancription factors	PPARG	208510_s_at							
Nuclear receptors & Trancription factors	PPARGC1A	219195_at	1569141_a_at						
Nuclear receptors & Trancription factors	PPARGC1B	232181_at	1553639_a_at	1555282_a_at	1563943_at				
Nuclear receptors & Trancription factors	PPRC1	203737_s_at							
Nuclear receptors & Trancription factors	PTGES3	200627_at							
Nuclear receptors & Trancription factors	RARA	203749_s_at	203750_s_at	211605_s_at	1565358_at				
Nuclear receptors & Trancription factors	RARB	205080_at	208412_s_at	208530_s_at	217020_at				
Nuclear receptors & Trancription factors	RARG	204188_s_at	204189_at	217178_at					
Nuclear receptors & Trancription factors	RXRA	202426_s_at	202449_s_at						
Nuclear receptors & Trancription factors	RXRB	209148_at	215098_at	215099_s_at					
Nuclear receptors & Trancription factors	RXRG	205954_at							
Nuclear receptors & Trancription factors	THRA	1316_at	204100_at	214883_at	35846_at				
Nuclear receptors & Trancription factors	THRB	207044_at	228716_at	229657_at					
Nuclear receptors & Trancription factors	TRIP11	209778_at	236160_at						
Nuclear receptors & Trancription factors	VDR	204253_s_at	204254_s_at	204255_s_at	213692_s_at				
Other genes	BLMH	202179_at							
Other genes	CRABP1	205350_at	1563897_at						
Other genes	CRABP2	202575_at							
Other genes	CYB5A	215726_s_at	217021_at						
Other genes	DCD	1553946_at							
Other genes	DCK	203302_at							
Other genes	DEFA1 : DEFA1B : DEFA3	205033_s_at							
Other genes	DEFA4	207269_at							
Other genes	DEFA5	207529_at							
Other genes	DEFA6	207814_at							
Other genes	DHDH	231416_at							
Other genes	GNLY	205495_s_at	37145_at						
Other genes	GZMA	205488_at							
Other genes	GZMB	210164_at							
Other genes	HAMP	220491_at							
Other genes	HTN3	206786_at							
Other genes	MT1M	217546_at							
Other genes	MT3	205970_at							
Other genes	MT4	217395_at							
Other genes	MTHFR	206800_at	217070_at	217071_s_at	226929_at	239035_at			
Other genes	NCEH1	225847_at							
Other genes	POR	208928_at							
Other genes	RBP1	203423_at	239782_at						
Other genes	RBP2	231734_at							
Other genes	S100A12	205863_at							
Other genes	SETD4	219482_at	230899_at						
Other genes	STX2	207346_at	213434_at						
Other genes	TP53	201746_at	211300_s_at						
Other genes	TXN	208864_s_at	216609_at						
Other genes	TXN2	209077_at	209078_s_at						
